# Longitudinal study of diagnostic sample matrices for respiratory pathogens on Irish farrow-to-finish pig herds

**DOI:** 10.1186/s13620-026-00333-7

**Published:** 2026-01-22

**Authors:** Rose Mary Fitzgerald, Edgar García Manzanilla, Julia Adriana Calderón Díaz, Patrick John Collins, John Moriarty, Hugh McGlynn, Helen O’Shea

**Affiliations:** 1 Bio-Explore, Department of Biological Sciences, Munster Technological University, Bishopstown, Cork, T12 P928 Republic of Ireland; 2 Pig Development Department, Teagasc Animal and Grassland Research and Innovation Centre, Moorepark, Fermoy, Co. Cork, P61 C996 Republic of Ireland; 3 School of Veterinary Medicine, University College Dublin, Belfield, Dublin 4, D04 V1W8 Republic of Ireland; 4 Veterinary Sciences Division, Agri-Food and Biosciences Institute, Stormont, Belfast, BT4 3SD Northern Ireland; 5 Cental Veterinary Research Laboratory, Department of Agriculture, Food and the Marine Laboratories, Co. Kildare, Backweston, Celbridge, W23 X3PH Republic of Ireland

**Keywords:** Actinobacillus pleuropneumoniae, Disease surveillance, Mycoplasma hyopneumoniae, Nasal swabs, Oral fluids, Porcine reproductive and respiratory syndrome virus, Influenza A virus, Veterinary diagnostics

## Abstract

**Background:**

Porcine respiratory disease complex (PRDC) involves multifactorial pathogen interactions that complicate surveillance strategies in commercial pig production. Current individual animal sampling methods present logistical constraints and animal welfare concerns. This longitudinal study evaluated the antibody responses and diagnostic efficacy of matched antemortem sample specimens (serum, nasal and pen-based oral fluids) for detecting *Actinobacillus pleuropneumoniae* (App), *Mycoplasma hyopneumoniae* (Mhyo), porcine reproductive and respiratory syndrome virus (PRRSV) and influenza A virus (IAV) across all production stages from weaning to slaughter (5 to 24 weeks of age) in seven Irish farrow-to-finish herds.

**Results:**

High-herd seroprevalence was observed at all five time-points, with concurrent antibody detection for the targeted respiratory pathogens occurring in 27.2% [95% confidence interval (CI) = 24.4–30.2%] of individual samples and 64.7% (95% CI = 46.5–80.3%) at herd level. Nucleic acid co-detection from two pathogens occurred in 11.1% of oral fluid and 15.3% of nasal swab samples, indicating widespread co-infection patterns. McNemar’s test demonstrated significant discordance between pathogen-specific immunoassays and PCR methodologies across all matrices (*p* < 0.05). Cohen’s kappa (κ) analysis revealed pathogen-specific agreement patterns. Slight matrix concordance was observed between nasal swabs and oral fluids for App (κ = 0.177) and Mhyo (κ = 0.135), while substantial agreement was demonstrated for PRRSV-1 (κ = 0.752) and IAV (κ = 0.629). Detection of PRRSV-1 showed moderate agreement between serum and nasal swabs (κ = 0.570) or oral fluids (κ = 0.575). Pathogen-specific detection peaks varied by agent and sample matrix with peaks for App and IAV at 5 weeks of age, Mhyo during finishing stages and extended PRRSV-1 detection window (10–19 weeks of age) in oral fluids. Prevalence detection rates were consistently equivalent or superior in oral fluids compared to nasal swabs, with significantly higher PRRSV-1 detection in oral fluids versus serum.

**Conclusions:**

Quantitative analysis validates oral fluid sampling as a diagnostically robust alternative to conventional matrices. The documented pathogen-specific temporal patterns and matrix-specific performance data provide evidence-based parameters for refining surveillance protocols. Statistical validation of oral fluid efficacy, combined with operational and welfare advantages, supports adoption of oral fluid sampling for comprehensive PRDC monitoring to enhance early pathogen detection, improve diagnostic accuracy, boost production efficiency and assist herd health management decisions in intensive pig production systems.

## Background

Respiratory diseases represent one of the most significant infectious health challenges in global pig production, with their prevalence exacerbated by intensive production systems that create conditions conducive to pathogen transmission and persistence [1–3]. Respiratory disease is multifactorial, involving polymicrobial infections [1, 2, 4], environmental stressors and management [1, 5], with economic consequences [1, 6–10].

In Ireland, herd-level serology from 56 farrow-to-finish farms showed high concurrent seroprevalence rates of major porcine respiratory disease complex (PRDC) agents [influenza A virus (IAV); 78.6%, porcine reproductive and respiratory syndrome virus (PRRSV); 50%, *Mycoplasma hyopneumoniae* (Mhyo); 74.1% and *Actinobacillus pleuropneumonia* (App); 98.2%] [11]. This is reflective of patterns observed internationally [8]. Slaughter surveillance in the same cohort recorded mean within-farm prevalences of pleurisy of 13%, predominantly classified as moderate to severe and pneumonia-like lesions recorded at 11%, with approximately 5.8% with low lung surface affected. Influenza A virus seropositivity was associated with pleurisy, while Mhyo seropositivity was linked with pneumonia and pericarditis, highlighting the complex aetiologies and clinical relevance of these infections in Irish herds [11].

Porcine respiratory disease complex predominantly manifests in post-weaning grow-finisher pigs [2], coinciding with the natural decline in maternally derived lactogenic immunity [12] and increased disease pressure from commingling of pigs from different litters [8, 13]. This temporal pattern underscores the critical vulnerability period when passive immunity wanes before adaptive immune responses can provide adequate protection. Strategic vaccination programs provide multiple benefits, including enhanced immunity through improved colostrum quality, superior post-vaccinal antibody responses, reduced pathogen loads, diminished clinical manifestations and improved overall animal performance [4, 14], yet Irish vaccination rates vary considerably, with 73.2% for Mhyo, 42.9% for PRRSV, 39.9% for IAV, and only 8.9% for App [11]. Despite the implementation of vaccination, proactive disease surveillance serves as the cornerstone of effective disease management, enabling early pathogen detection and facilitating rapid implementation of control measures, including treatment protocols, animal isolation, disease monitoring and elimination strategies [15–17].

Routine surveillance typically targets diseases known or suspected to be present through seroprevalence studies or slaughterhouse monitoring to establish baseline health status or confirm freedom from disease within herds [16, 18–20]. The selection of appropriate diagnostic sample matrices fundamentally influences the effectiveness of surveillance programs, with antemortem matrices differing in performance and feasibility. Traditional antemortem sampling for respiratory disease diagnosis utilises serum, nasal swabs and tracheal swabs, each offering specific advantages for different pathogen detection scenarios. Serum samples enable antibody detection and assessment of systemic pathogen presence, particularly for agents such as PRRSV [19, 21] and are influenced by variable seroconversion timings, while nasal swabs are optimised for detecting upper respiratory tract pathogens, including App [22]. Individual nasal swabs represent the antemortem gold-standard for IAV direct detection during acute infection, outperforming oral fluids in direct comparison [23]. Despite lower individual sensitivity, oral fluids provide an extended IAV detection window (approximately 2–4 weeks during episodes) and offer practical advantages and feasibility for repeated pen-level surveillance, whereas PRRSV and Mhyo detection is more intermittent across pens and time-points [24, 25]. The most sensitive sampling method for early detection of Mhyo is trachea-bronchial swabbing, especially during the early stages of infection [26–28]. Oral fluids produce approximately double the probability of detection of Mhyo as the same number of serum samples at 35 days post-infection (DPI), with the collection of tracheal samples requiring more time and animal restraint than serum or oral fluids [24, 27, 29] and impractical for routine herd monitoring.

Conventional sampling methods present significant challenges, especially within longitudinal studies, including animal stress, labour intensity and requirements for specialised personnel training [19, 24, 30]. Oral fluids, comprising of saliva and mucosal transudate [19, 24, 31–33], have emerged as an innovative, feasible alternative [34]. The voluntary nature of oral fluid collection eliminates animal restraint requirements while enabling safe sample collection by animal caretakers, thereby reducing biosecurity risks [19, 34]. Pen-based oral fluids represent a pooled sampling strategy [19, 35] and enable cost-effective screening of large populations through sequential sampling at different production stages [17]. Strategic fixed spatial sampling across equidistant pens along the length of a barn enhances pathogen detection probability by accounting for pig-to-pig and pen-to-pen transmission patterns [17].

Given the polymicrobial nature of PRDC, longitudinal molecular diagnostic data enables the identification of circulating primary pathogens [35] and their spatial distribution at specific time-points (t/p) [35]. However, pathogen detection does not necessarily correlate with clinical disease manifestation, emphasising the importance of integrating molecular results with clinical observations. Despite the recognised importance of PRDC surveillance, limited data exists regarding concurrent assessment of multiple pathogens from matched sample matrices from the same antemortem animal source [20, 35–37]. This lack of integrated, comparative diagnostic data restricts the understanding of pathogen dynamics, optimal detection windows and relative performance of different sample types for PRDC surveillance. The Irish burden of PRDC and documented associations between pathogens and lesions further motivate proactive, serial surveillance design in farrow-to-finish systems [11].

The objective of this study was to address this knowledge gap through a comprehensive longitudinal evaluation of diagnostic sample matrix performance for four prominent PRDC pathogens in Irish farrow-to-finish production systems. Matched samples (serum and nasal swabs) and pen-based oral fluids were collected from the same weekly batch at five time-points across the complete production cycle from weaning to slaughter (5 to 24 weeks of age) on seven Irish commercial herds. Through concurrent application of pathogen-specific serological and molecular diagnostic methods, this integrated approach aimed to: (i) characterise temporal patterns of pathogen detection and antibody responses throughout production stages, (ii) quantify diagnostic concordance between sample matrices and detection methods, (iii) document co-circulation and co-detection patterns among PRDC pathogens, and (iv) establish evidence-based recommendations for optimal surveillance strategies that balance diagnostic performance with practical feasibility for commercial application. The design aligns with Irish herd epidemiology, maximising detection opportunities across pathogens with different shedding windows and balancing diagnostic performance with practicality for on-farm surveillance.

## Methods

A longitudinal study was conducted on seven commercial Irish farrow-to-finish pig herds (designated Herds A to G), between November 2016 to June 2017, to investigate respiratory pathogen circulation patterns using multiple sample matrices. This research formed part of a larger investigation examining the prevalence of respiratory disease, associated risk factors, and relationships with performance, welfare, and antimicrobial use on Irish pig farms.

Herd recruitment was conducted through the Teagasc e-Profit Monitor system, a comprehensive recording platform that captures detailed productive and economic data from over 52% of Ireland’s national commercial sow herd (representing 77,000 sows in 2017), and on their participation of a biosecurity questionnaire completed as part of a large cross-sectional study including 56 Irish farms [38]. Participating herds were selected, based on voluntary participation in the monitoring system and met the following inclusion criteria: (i) operation of a weekly farrowing batch system, (ii) farrow-to-finish production system, (iii) reported historical respiratory disease challenges and (iv) implementation of all-in-all-out (AIAO) management practices with no acute disease outbreaks in the 6 months immediately preceding study enrollment or during the study duration of ~ 24 weeks, from birth (i.e. selection) to slaughter.

The recruited herds represented a diverse range of production scales, with sow herd sizes ranging from 650 to 2,354 sows (mean ± standard deviation: 1,146 ± 832 sows; data not available for Herd B). Vaccination protocols for respiratory pathogens were documented prior to study commencement (Table 1). Preventative vaccination strategies varied among herds, with bacterial pathogen vaccines (App and Mhyo) administered to piglets, while viral pathogen vaccines (PRRSV and IAV) were administered to breeding stock (sows and gilts). Notably, Herd G operated without vaccination protocols for any of the four target pathogens.

**Table 1:** Vaccination protocol^*a*^ information of seven Irish farrow-to-finish commercial herds for four respiratory pathogens of interest

	Herd vaccination preventative protocols for the respiratory pathogen of interest				
Herd ID	App	Mhyo	PRRSV	IAV	
Herd A	No	Yes: piglets	Yes: sows	Yes: sows	
		25 days old	60 days after breeding and 7 days after farrowing	repeat after every 4-month interval	
			Yes: gilts	Yes: gilts	
			2-dose vaccination at selection and 60 days after breeding	repeat after every 4-month interval	
Herd B	No	Yes: piglets	Yes: sows	Yes: sows	
		14 and 26 days old	80 days after breeding and 5 days after farrowing	repeat after every 6-month interval	
			Yes: gilts	Yes: gilts	
			80 days after breeding and 5 days after farrowing	repeat after every 6-month interval	
Herd C	Yes: piglets	Yes: piglets	Yes: sows	Yes: sows	
	21 and 35 days old	14 and 24 days old	60 days after breeding and 6 days after farrowing	6 weeks before farrowing	
			Yes: gilts	Yes: gilts	
			21 days after breeding and 6 days after farrowing	3 weeks before farrowing	
Herd D	No	Yes: piglets	Yes: sows	No	
		10 and 28 days old	60 days after breeding		
			Yes: gilts		
			before breeding and 60 days after breeding		
Herd E	No	Yes: piglets	No	Yes: sows	
		10 and 28 days old		3 weeks before farrowing	
				Yes: gilts	
				6- and 3-weeks before breeding	
Herd F	No	Yes: piglets	No	No	
		21–24 days old			
Herd G	No	No	No	No	

Vaccination protocols^*a*^ for targeted respiratory pathogens (*Actinobacillus pleuropneumonia* (App), *Mycoplasma hyopneumoniae* (Mhyo), porcine reproductive and respiratory syndrome virus (PRRSV) and influenza A virus (IAV)

### Animal management

On each of the seven participating herds, on average, 36 piglets were randomly selected within 24 to 72 h post-farrowing and individually tagged for longitudinal tracking until slaughter. Systematic random sampling was employed, with piglets selected from multiple litters across the weekly farrowing batch to ensure balanced representation across sow parity groups (i.e. primiparous, parity 2–3, and parity 4+) and similar gender distribution (i.e. male and female piglets). All study animals underwent weaning at approximately 28 days of age, consistent with standard Irish commercial practice [39], regardless of individual body weight. The selected pigs remained integrated with their production cohorts throughout the study, advancing through conventional production stages with non-study animals within the same farrowing batch. This approach maintained normal stocking densities and social dynamics while minimising disruption to routine herd operations.

The Irish pig production system follows a standardised age-based batch progression through distinct housing phases: weaner stage (~ 5–9 weeks of age), weaner stage 2 (~ 10–13 weeks of age) and finisher stage (~ 14–24 weeks of age) [39]. Each participating herd maintained its established management protocols throughout the study period, including housing configurations which varied between herds and typically comprised either multiple houses per production stage or single houses subdivided into multiple pens. Herd-specific management practices, including feed regimens, vaccination schedules and environmental controls, continued unchanged to preserve the representativeness of routine commercial conditions and ensure external validity of the study findings.

### On-site sample collection

The longitudinal sampling protocol consisted of five time-points (t/p) per animal, commencing at t/p 1 (5 weeks of age, one-week post-weaning) and continued at approximately 4-week intervals at t/p’s 2, 3, 4 and 5, corresponding to 10, 15, 19 and 24 weeks of age, respectively. This sampling framework generated five collection opportunities per pig across the production cycle. From each herd, ten pigs from the tagged cohort provided paired nasal swabs from t/p 1 through t/p 4. Pen-based oral fluids were collected from both pens where the tagged animals were housed and from pens adjacent to the study cohort, reflecting pig-to-pig and pen-to-pen transmission patterns [17, 27], using the methodology described by Prickett et al. [33]. No samples were collected from Herd E at t/p 1.

Individual blood samples were obtained from each tagged pig via the jugular venipuncture using sterile technique [Vacutainer^®^ Blood Collection Tubes, (Becton, Dickinson, United Kingdom (UK) Ltd., Berkshire, UK)]. Paired nasal swabs were collected using sterile polyester-tipped swabs (Thermo Scientific™ Sterilin™, Fisher Scientific, Leicestershire, UK). Each swab was inserted 2–3 cm into the nostril, rotated gently against the nasal mucosa for 10–15 s and immediately placed in viral transport media. Pen-based oral fluids were obtained by securely suspending a length of ~ 1.6 cm, 3-stranded, twisted, unbleached cotton rope in selected pens at pig shoulder height in areas free from feed and water contamination. Ropes remained accessible for 20–30 min to allow natural chewing behaviour from the pigs, maintaining a ratio of one rope per 25 pigs (maximum) as recommended by Prickett et al. [33].

### Sample processing and storage

All samples were transported on ice and stored at 4 °C until initial processing (within 24 h of collection). Serum was separated from clotted whole blood by centrifugation at 2,800 revolutions per minute (RPM) for 5 min at 4 °C and aliquoted into individually pre-labelled, anonymised cryovials (SARSTEDT^®^, Nümbert, Germany). Nasal swabs were transported in pre-filled bijou containers (Thermo Scientific™ Sterlin™, Fisher Scientific, Leicestershire, UK) containing viral transport medium, centrifuged at 2,800 RPM for 5 min at 4 °C, and stored in individually pre-labelled, anonymised bijou containers. Cotton ropes were removed from the pen and individually sealed in sterile transparent bags (Merck KGaA, Darmstadt, Germany) and manually compressed to extract the oral fluid. The bag corner was aseptically cut, and the extracted fluid was dispensed into pre-labelled 50 mL screw-cap tubes (SARSTEDT^®^, Nümbert, Germany). Oral fluid samples were subjected to centrifugation at 2,800 RPM for 30 min at 4 °C to remove particulate matter [40]. All processed samples were stored at -80 °C until laboratory analysis or additional processing was performed.

### Serological analysis

Herd-level health status was assessed through serological testing of all individual serum samples (*n* = 936) across all t/p’s using commercially available pathogen-specific (App, Mhyo, PRRSV and IAV) enzyme-linked immunosorbent assays (ELISA) (IDEXX Europe B.V., Hoofddorp, The Netherlands). The following IDEXX ELISA kits were employed with their respective performance characteristics: App: ApxIV Ab Test (sensitivity 13–74%, specificity 90–100% [41]. Manufacturer-reported performance: sensitivity 97.8%, specificity 100%) to detect the ApxIV toxin, which is produced during infection by all known App serotypes [20, 37, 42], enabling differentiation between infected and vaccinated animals; Mhyo: HerdChek^®^ *Mycoplasma hyopneumoniae* Antibody Test (sensitivity 55.7%, specificity 98.8% [43]. Manufacturer-reported performance: sensitivity 89.4%, specificity 99.7%); PRRSV: PRRS X3 *Ab* Test (sensitivity 100%, specificity 100% [44]. Manufacturer-reported performance: sensitivity 98.8%, specificity 99.9%) to detect both European (PRRSV-1) and North American (PRRSV-2) species; IAV; Influenza A Ab Test (sensitivity 82.9%, specificity 76.7% [45]. Manufacturer-reported performance: sensitivity 95.3%, specificity 99.6%) to detect H1N1, H1N2 and H3N2 serotypes from swine sera. All assays were performed according to the manufacturer’s protocols with appropriate positive in-house controls and kit-supplied positive and negative controls incorporated during the serodiagnostic testing. Antibody quantification was achieved through colourimetric detection using spectrophotometry on a TECAN Sunrise™ microplate reader (Tecan Group Ltd., Männedorf, Switzerland), incorporating TECAN Magellan™ data analysis software v7.1 (Tecan Group Ltd., Männedorf, Switzerland). An important limitation to note is that the Mhyo, PRRSV and IAV commercial kits cannot distinguish between vaccination-induced and infection-derived antibody responses.

Individual serum samples were classified as seropositive based on sample ratio values: App ≥ 0.50, Mhyo > 0.40, PRRSV ≥ 0.40 and IAV < 0.60. Suspect ELISA results for App and Mhyo were considered negative in the absence of repeat testing [37]. Herd-level classification employed herd-specific cut-off prevalence thresholds to minimise false-positive classification due to individual seropositive animals. Herds A, B, C and E (*n* = 24 pigs per herd) were classified as positive when > 4.2% of animals were seropositive (> 1/24), while Herds D, F and G (*n* = 32 pigs per herd) required > 3.1% seropositive animals (> 1/32) for positive classification. These thresholds account for the high specificity (99.6–100%) of the IDEXX assays employed.

### Polymerase chain reaction analysis

Nucleic acid extraction was performed on 200 µl aliquots of all processed samples: serum (*n* = 936; all t/p’s), paired nasal swabs (*n* = 292) and oral fluids (*n* = 147) collected from t/p 1 through t/p 4, utilising the Roche MagNA Pure LC Total Nucleic Acid Isolation Kit (Roche Diagnostics Ltd., Burgess Hill, West Sussex, UK) on the Roche MagNA Pure LightCycler 2.0 Instrument (Roche Diagnostic Ltd., UK) according to manufacturer’s specifications. Positive and negative extraction controls were included in each run.

For viral pathogen detection (PRRSV and IAV), real-time quantitative reverse transcriptase PCR (qRT-PCR) using the AgPath-ID™ One-Step RT-PCR kit (ThermoFisher Scientific, Applied Biosystems™, Waltham, Massachusetts, USA) was employed. Species-specific primer/probe sets targeting the open reading frame 7 region distinguished between PRRSV-1 (European) and PRRSV-2 (North American) species [46]. For IAV detection, a primer/probe set targeting the conserved matrix gene of influenza A viruses was employed [47]. For bacterial pathogen detection, App and Mhyo utilised the quantitative real-time Quantitect™ SYBR^®^ Green PCR kit (Qiagen Ltd., Manchester, UK). A primer set targeting the *apx*IVA gene [48] was employed for App detection, while Mhyo detection used a primer set described by Strait et al. [49] targeting the mhp165 gene.

All PCR reactions were performed on the Roche LightCycler^®^ 96 Instrument (Roche Diagnostics Ltd., UK) according to the manufacturer’s protocols. Samples were analysed in duplicate with appropriate inter-run and extraction controls. Data analysis employed LightCycler^®^ Application Software V1.1. Samples were considered PCR-positive when samples yielded a cycle threshold (Ct) value ≤ 40 in replicate wells. Results were further stratified by signal strength: ‘strong positive’ (Ct ≤ 37 in replicate wells) and ‘weak positive’ (Ct > 37 and ≤ 40 in replicate wells). Herd-level pathogen presence was determined when at least one sample from any matrix (serum, nasal swab or oral fluid) tested positive at a given t/p.

### Data analyses

All statistical analyses were conducted using R statistical software version 4.2.1 [50]. Data visualisation and manipulation were performed using the *ggplot2* package version 5.1 [51] and *dplyr* package version 1.1.2 [52] within the tidyverse framework, respectively.

Herd-level positivity at each t/p was defined as detection of pathogen-specific antibodies, following the application of herd-prevalence cut-off criteria or direct pathogen detection by PCR from any collected sample matrix (serum, nasal swabs or oral fluids). This binary classification system enabled comparative assessment across different detection methods and sample types. The diagnostic performance of each sample matrix (serum, nasal swabs and oral fluids) was compared for each respiratory pathogen using multiple analytical approaches. The equality of detection proportions between sample matrices was assessed using McNemar’s test for paired nominal data, appropriate for comparing matched samples from the same animals or pens. Statistical significance was set at *p <* 0.05. Inter-matrix diagnostic agreement was evaluated using Cohen’s kappa coefficient (κ), the standard measure for diagnostic concordance [53]. Specific comparisons included: the strength of agreement of oral fluid versus nasal swabs by PCR for all four pathogens; serum versus oral fluid or nasal swabs by PCR specifically for PRRSV-1 detection; and ELISA versus PCR results within each sample matrix for each pathogen. Cohen’s kappa values were interpreted according to established criteria. The interpretation ranges for the strength of agreement are outlined as: κ ≤ 0.00 (no agreement); κ = 0.01–0.20 (slight agreement); κ = 0.21–0.40 (fair agreement); κ = 0.41–0.60 (moderate agreement); κ = 0.61–0.80 (substantial agreement); and κ = 0.81-1.00 (almost perfect agreement) [54].

## Results

### Serological

The number of seropositive herds and within-herd prevalence data are presented in Tables 2 and 3, respectively. Universal seroconversion of App occurred across all seven herds during the first weaner stage, with 100% of herds testing positive at t/p 1 (5 weeks of age). Individual animal seroprevalence was highest at this initial t/p, with 97.6% [95% confidence interval (CI) = 94.0-99.3%] of samples testing positive. Seroprevalence subsequently declined but remained substantial throughout the production cycle: 67.2% (95% CI = 60.1–73.8%) at t/p 2, 74.0% (95% CI = 67.1–80.0%) at t/p 3, 86.5% (95% CI = 80.8–91.0%) at t/p 4, and 93.2% (95% CI = 88.7–96.3%) at t/p 5, corresponding to 10, 15, 19, and 24 weeks of age, respectively.

**Table 2:** Longitudinal serological results for seven Irish farrow-to-finish herds for four respiratory pathogens of interest

Pathogen,	age	t/p	Seropositive herds	Seropositive herds			
Stage	(wks)			(With-in herd prevalence subdivided into percentage categories)			
				3.1/4.2–25^**^	26–50	51–75	76–100
App							
Weaner 1^*^	5	1	6 (100%)	-	-	-	6 (100%)
Weaner 2	10	2	7 (100%)	1 (14%)	1 (14%)	2 (29%)	3 (43%)
Finishers	15	3	7 (100%)	1 (14%)	1 (14%)	1 (14%)	4 (57%)
Finishers	19	4	7 (100%)	1 (14%)	-	-	6 (86%)
Slaughter	24	5	7 (100%)	-	-	1 (14%)	6 (86%)
Mhyo							
Weaner 1^*^	5	1	5 (83%)	2 (33%)	-	2 (33%)	1 (17%)
Weaner 2	10	2	6 (86%)	-	-	3 (43%)	3 (43%)
Finishers	15	3	6 (86%)	-	-	2 (29%)	4 (57%)
Finishers	19	4	6 (86%)	-	-	-	6 (86%)
Slaughter	24	5	6 (86%)	-	-	-	6 (86%)
PRRSV							
Weaner 1^*^	5	1	4 (67%)	-	1 (17%)	2 (33%)	1 (17%)
Weaner 2	10	2	4 (57%)	4 (57%)	-	-	-
Finishers	15	3	4 (57%)	-	-	-	4 (57%)
Finishers	19	4	4 (57%)	-	-	-	4 (57%)
Slaughter	24	5	4 (57%)	-	-	-	4 (57%)
IAV							
Weaner 1^*^	5	1	6 (100%)	2 (33%)	2 (33%)	-	2 (33%)
Weaner 2	10	2	6 (86%)	-	1 (14%)	4 (57%)	1 (14%)
Finishers	15	3	6 (86%)	-	2 (29%)	1 (14%)	3 (43%)
Finishers	19	4	6 (86%)	-	1 (14%)	1 (14%)	4 (57%)
Slaughter	24	5	6 (86%)	-	1 (14%)	2 (29%)	3 (43%)

Longitudinal serological results for seven Irish farrow-to-finish herds expressed as a percentage of the studied herds at each time-point (t/p) for four respiratory pathogens [*Actinobacillus pleuropneumonia* (App), *Mycoplasma hyopneumoniae* (Mhyo), porcine reproductive and respiratory syndrome virus (PRRSV) and influenza A virus (IAV)]

^*^Six of seven participating herds were sampled at t/p 1

^**^Herd prevalence cut-off was established at 3.1% for Herds A, B, C and E and 4.2% for Herds F and G as a single seropositive animal was interpreted as a false positive (refer to text)

**Table 3:** Antibody-positive samples per herd and within-herd antibody prevalence for respiratory pathogens on Irish farrow-to-finish herds

			Herd Identification							
Pathogen,	Age	t/p	Herd A	Herd B	Herd C	Herd D	Herd E	Herd F	Herd G	Total percentage
production stage	(wks)		Number of antibody-positive samples and within-herd antibody prevalence (%)							seropositivity
App										
Weaner 1*	5	1	24(100%)	23(95.8%)	24(100%)^*^	32(100%)	ns	30(93.8%)	31(96.9%)	164(97.6%)
Weaner 2	10	2	23(95.8%)	10(41.7%)	23(95.8%)^*^	30(93.8%)	18(75%)	8(25%)	17(53.1%)	129(67.2%)
Finishers	15	3	24(100%)	9(37.5%)	23(95.8%)^*^	29(90.6%)	3(12.5%)	24(75%)	30(93.8%)	142(74.0%)
Finishers	19	4	24(100%)	24(100%)	24(100%)^*^	32(100%)	2(8.3%)	30(93.8%)	30(93.8%)	166(86.5%)
Slaughter	24	5	24(100%)	24(100%)	24(100%)^*^	32(100%)	13(54.2%)	32(100%)	30(93.8%)	179(93.2%)
Mhyo										
Weaner 1*	5	1	17(70.8%)^*^	14(58.3%)^*^	3(12.5%)^*^	28(87.5%)^*^	ns	2(6.2%)^*^	0(0%)	64(38.1%)
Weaner 2	10	2	17(70.8%)^*^	15(62.5%)^*^	22(91.7%)^*^	29(90.6%)^*^	19(79.2%)^*^	23(71.9%)^*^	0(0%)	125(65.1%)
Finishers	15	3	17(70.8%)^*^	20(83.3%)^*^	21(87.5%)^*^	31(96.9%)^*^	16(66.7%)^*^	31(96.9%)^*^	0(0%)	136(70.8%)
Finishers	19	4	24(100%)^*^	24(100%)^*^	23(95.8%)^*^	31(96.9%)^*^	23(95.8%)^*^	32(100%)^*^	0(0%)	157(82.2%)
Slaughter	24	5	24(100%)^*^	24(100%)^*^	24(100%)^*^	32(100%)^*^	24(100%)^*^	32(100%)^*^	1(3.1%)	161(83.4%)
PRRSV										
Weaner 1*	5	1	16(66.7%)^†^	17(70.8%)^†^	19(79.2%)^†^	16(50%)^†^	ns	0(0%)	0(0%)	68(40.5%)
Weaner 2	10	2	6(25%)^†^	2(8.3%)^†^	5(20.8%)^†^	7(21.9%)^†^	0(0%)	0(0%)	0(0%)	20(10.4%)
Finishers	15	3	24(100%)^†^	21(87.5%)^†^	23(95.8%)^†^	32(100%)^†^	1(4.2%)	0(0%)	0(0%)	101(52.6%)
Finishers	19	4	23(95.8%)^†^	24(100%)^†^	24(100%)^†^	32(100%)^†^	0(0%)	0(0%)	0(0%)	103(53.6%)
Slaughter	24	5	24(100%)^†^	24(100%)^†^	24(100%)^†^	32(100%)^†^	1(4.2%)	0(0%)	1(3.1%)	106(55.2%)
IAV										
Weaner 1*	5	1	24(100%)^†^	12(50%)^†^	23(95.8%)^†^	15(46.9%)	ns	2(6.2%)	6(18.8%)	82(48.8%)
Weaner 2	10	2	14(58.3%)^†^	21(87.5%)^†^	18(75%)^†^	22(68.8%)	11(45.8%)^†^	22(68.8%)	0(0%)	108(56.3%)
Finishers	15	3	16(66.7%)^†^	19(79.2%)^†^	19(79.2%)^†^	11(34.4%)	23(95.8%)^†^	15(46.9%)	0(0%)	103(53.6%)
Finishers	19	4	24(100%)^†^	20(83.3%)^†^	19(79.2%)^†^	32(100%)	17(70.8%)^†^	1(43.8%)	0(0%)	126(65.6%)
Slaughter	24	5	22(91.7%)^†^	21(87.5%)^†^	15(62.5%)†	27(84.4%)	15(62.5%)^†^	13(40.6%)	0(0%)	113(58.9%)

Number of antibody-positive samples per herd and within-herd antibody prevalence across 7 Irish farrow-to-finish herds at each sampling time-point (t/p) (*n* = 5) throughout pig production stages (weaner and finishers) and at slaughter. The serology samples (*n* = 936) were analysed using commercially available pathogen-specific (*Actinobacillus pleuropneumonia* (App), *Mycoplasma hyopneumoniae* (Mhyo), porcine reproductive and respiratory syndrome virus (PRRSV) and influenza A virus (IAV) IDEXX enzyme-linked immunosorbent assays. No samples (ns) were collected or available from Herd E at t/p 1

^†^Pathogen-specific vaccination administered to sows and gilts

^*^Pathogen-specific vaccination administered to piglets

A vaccination-associated pattern emerged for Mhyo seropositivity. All six vaccinated herds achieved 100% seroprevalence at slaughter (t/p 5), while Herd G, the only unvaccinated herd, remained seronegative throughout the study period (Table 3). Among the vaccinated herds, seroprevalence increased progressively from 38.1% (95% CI = 30.7–45.9%) at t/p 1 (5 weeks of age) to peak levels of 83.4% (95% CI = 77.9–88.8%) at t/p 5 (24 weeks of age).

The PRRSV seroprevalence peaked at slaughter, reaching 55.2% (95% CI = 47.9–62.4%) at t/p 5 (24 weeks of age), with four herds achieving 100% seroprevalence at this time-point. Notably, all seropositive herds had implemented PRRSV vaccination programs (Table 1). Influenza A virus exhibited considerable inter-herd variation in seroprevalence, with antibody levels observed consistently exceeding 54% during the finisher stages and at slaughter. Specific seroprevalence values were 53.6% (95% CI = 46.3–60.9%) at t/p 3 (15 weeks of age), 65.6% (95% CI = 58.4–72.3%) at t/p 4 (19 weeks of age) and 58.9% (95% CI = 51.5–65.9%) at t/p 5 (24 weeks of age) (Table 3).

Simultaneous exposure to multiple respiratory pathogens was common, with antibodies for all four target pathogens detected in 27.2% [24.4–30.2%] of individual serum samples. At herd level, 64.7% [46.5–80.3%] of herds tested seropositive for all four investigated pathogens simultaneously. The level of concurrent seropositivity occurred at t/p 3 (15 weeks of age) and t/p 5 (24 weeks of age), when five of the seven herds (71.4%) demonstrated simultaneous seropositivity for all targeted respiratory pathogens (Table 3).

### Polymerase chain reaction

Within-herd pathogen prevalence as determined by PCR analysis of three sample matrices across all participating herds is presented in Table 4. From the analysed serum samples, PRRSV-1 was detected in 36 of 436 samples (8.3%), originating from four participating herds. Detection was concentrated in the weaner and early finishing stages, with peak prevalence occurring at t/p 2 (10 weeks of age) at 21.1% (95% CI = 13.2–31.0%). All serum samples tested negative for PRRSV-2, indicating exclusive circulation of the European strain within the study population.

**Table 4:** Pathogen detection results and within-herd prevalence for Irish farrow-to-finish herds using multiple sample matrices

		Herd Identification							
Sample matrix	Respiratory pathogen	Herd A	Herd B	Herd C	Herd D	Herd E	Herd F	Herd G	Total percentage
Serum	PRRSV-1	5/58 (8.6%)^†^	12/69 (17.4%)^†^	9/99 (9.1%)^†^	10/56 (17.9%)^†^	0/54 (0.0%)	0/50 (0.0%)	0/50 (0.0%)	36/436 (8.3%)
	PRRSV-2	0/58 (0.0%)	0/69 (0.0%)	0/99 (0.0%)	0/56 (0.0%)	0/54 (0.0%)	0/50 (0.0%)	0/50 (0.0%)	0/436 (0.0%)
Nasal	App	6/37 (16.2%)	4/52 (7.7%)	5/49 (10.2%)^*^	0/46 (0.0%)	1/38 (2.6%)	2/40 (5.0%)	7/30 (23.3%)	25/292 (8.6%)
	Mhyo	0/37 (0.0%)^*^	1/52 (1.9%)^*^	0/49 (0.0%)^*^	0/46 (0.0%)^*^	0/38 (0.0%)^*^	0/40 (0.0%)^*^	0/30 (0.0%)	1/292 (0.3%)
	PRRSV-1	0/37 (0.0%)^†^	3/52 (5.8%)^†^	3/49 (6.1%)^†^	5/46 (10.9%)^†^	0/38 (0.0%)	0/40 (0.0%)	0/30 (0.0%)	11/292 (3.8%)
	PRRSV-2	0/37 (0.0%)	0/52 (0.0%)	0/49 (0.0%)	0/46 (0.0%)	0/38 (0.0%)	0/40 (0.0%)	0/30 (0.0%)	0/292 (0.0%)
	IAV	2/37 (5.4%)^†^	0/52 (0.0%)^†^	4/49 (8.2%)^†^	3/46 (6.5%)	1/38 (2.6%)^†^	0/40 (0.0%)	0/30 (0.0%)	10/292 (3.4%)
Oral fluid	App	0/12 (0.0%)	0/14 (0.0%)	0/36 (0.0%)^*^	0/16 (0.0%)	2/22 (9.1%)	0/23 (0.0%)	10/24 (41.7%)	12/147 (8.2%)
	Mhyo	1/12 (8.3%)^*^	1/14 (7.1%)^*^	0/36 (0.0%)^*^	2/16 (12.5%)^*^	3/22 (13.6%)^*^	2/23 (8.7%)^*^	0/24 (0.0%)	9/147 (6.1%)
	PRRSV-1	0/12 (0.0%)^†^	5/14 (35.7%)^†^	5/36 (13.9%)^†^	4/16 (25.0%)^†^	0/22 (0.0%)	0/23 (0.0%)	0/24 (0.0%)	14/147 (9.5%)
	PRRSV-2	0/12 (0.0%)	0/14 (0.0%)	0/36 (0.0%)	0/16 (0.0%)	0/22 (0.0%)	0/23 (0.0%)	0/24 (0.0%)	0/147 (0.0%)
	IAV	1/12 (8.3%)^†^	0/14 (0.0%)^†^	0/36 (0.0%)^†^	4/16 (25.0%)	0/22 (0.0%)^†^	0/23 (0.0%)	0/24 (0.0%)	5/147 (3.4%)

Pathogen detection results per herd and within-herd prevalence across 7 Irish farrow-to-finish herds throughout pig production stages (weaner and finishers) and at slaughter using polymerase chain reaction technologies on multiple sample matrices (serum, nasal swabs and oral fluids) for four target respiratory pathogens [*Actinobacillus pleuropneumonia* (App), *Mycoplasma hyopneumoniae* (Mhyo), porcine reproductive and respiratory syndrome virus (PRRSV-1; European species and PRRSV-2; North American species) and influenza A virus (IAV)]. Oral fluid and nasal swabs were collected at time-points 1-4; serum collected at all 5 time-points

^†^Pathogen-specific vaccination administered to sows and gilts

^*^Pathogen-specific vaccination administered to piglets

Nasal swab analysis revealed distinct detection patterns for each pathogen (Table 4). *Actinobacillus pleuropneumoniae* was detected from nasal swabs across all t/p’s with an overall prevalence of 8.6% and was present in six of the seven herds, with peak detection occurring at the latter weaner stage (t/p 2; 10 weeks of age), with 18.9% (95% CI = 11-29.4%) positivity with Ct values ranging from 25.1 to 37.9.

In contrast, Mhyo showed minimal detection from nasal swabs, with only one positive sample identified from Herd B at t/p 4 (Ct value: 33.8), which contrasts markedly with serological findings and oral fluid results. The detection pattern of PRRSV-1 mirrored that observed in serum, peaking at t/p 2 (10 weeks of age) with an 8.9% (95% CI = 3.6–17.4%) prevalence and Ct values ranging from 29.1 to 38.8, while all samples were negative for PRRSV-2.

The IAV RNA virus was detected exclusively during the weaner stages, with peak prevalence at t/p 1 (5 weeks of age) of 14.75% (95% CI = 7.0-26.2%) and Ct values ranging from 25.4 to 33.7. Sixty-eight percent of nasal swabs tested PCR-positive for at least one targeted respiratory pathogen, demonstrating high pathogen circulation, while co-detection occurred in 15.38% of samples, predominantly involving App and PRRSV-1.

Oral fluid samples demonstrated enhanced sensitivity for certain pathogens, with Ct values indicating successful pathogen detection. The highest detection of App nucleic acid from oral fluids was at t/p 1 with 28.1% (95% CI = 13.8–46.8%) positivity and Ct values ranging from 31.8 to 40.5. Contrasting with the nasal swab result, nine oral fluid samples tested PCR-positive for Mhyo, predominantly in latter t/p’s, with peak detection occurring at t/p 4 (19 weeks of age), with 18.8% (95% CI = 7.2–36.4%) prevalence and Ct values ranging from 32.1 to 39.5. The PRRSV-1 RNA was detected at consecutive t/p’s (2, 3 and 4) with decreasing prevalence of 18.6% (95% CI = 8.4–33.4%), 10% (95% CI = 2.5–23.7%) and 6.3% (95% CI = 0.8–20.8%), respectively, with Ct values ranging from 33.6 to 39.1, while all samples were negative for PRRSV-2.

The detection of IAV nucleic acid was limited to t/p 1 (5 weeks of age) on two herds (Herd A; 25% detection rate and Herd D; 100% detection rate), with Ct values ranging from 30.3 to 33.0. Fifty-six percent of oral fluid samples tested PCR-positive for at least one targeted pathogen, with Mhyo being the most frequently detected (29.6% of all samples), while co-detection occurred in 11.1% of samples, involving various pathogen combinations, including Mhyo/IAV, Mhyo/PRRSV-1 and Mhyo/App.

The comprehensive herd status determined by both ELISA and PCR techniques across different sample matrices for the four targeted respiratory pathogens is summarised in Table 5.

**Table 5:** Herd pathogen status determined by different diagnostic methodologies using multiple sample matrices

				Targeted Porcine Respiratory Pathogens																
				App			Mhyo			PRRSV-1				PRRSV-2				IAV		
Herd	Stage	Age	t/p	Serum	Nasal	Oral fluids	Serum	Nasal	Oral fluids	Serum	Serum	Nasal	Oral fluids	Serum	Serum	Nasal	Oral fluids	Serum	Nasal	Oral fluids
ID		(wks)		ELISA	PCR	PCR	ELISA	PCR	PCR	ELISA	PCR	PCR	PCR	ELISA	PCR	PCR	PCR	ELISA	PCR	PCR
A	Weaner 1	5	1	+	-	-	+	-	-	+	+	-	-	-	-	-	-	+	+	+
	Weaner 2	10	2	+	+	-	+	-	-	+	+	-	-	-	-	-	-	+	-	-
	Finisher	15	3	+	+	-	+	-	-	+	+	-	-	-	-	-	-	+	-	-
	Finisher	19	4	+	+	-	+	-	+	+	-	-	-	-	-	-	-	+	-	-
	Slaughter	24	5	+	ns	ns	+	ns	ns	+	-	ns	ns	-	-	ns	ns	+	ns	ns
B	Weaner 1	5	1	+	+	-	+	-	-	+	-	+	-	-	-	-	-	+	-	-
	Weaner 2	10	2	+	+	-	+	-	-	+	-	-	-	-	-	-	-	+	-	-
	Finisher	15	3	+	+	-	+	-	-	+	+	+	+	-	-	-	-	+	-	-
	Finisher	19	4	+	-	-	+	+	+	+	-	-	+	-	-	-	-	+	-	-
	Slaughter	24	5	+	ns	ns	+	ns	ns	+	-	ns	ns	-	-	ns	ns	+	ns	ns
C	Weaner 1	5	1	+	-	-	+	-	-	+	-	-	-	-	-	-	-	+	+	-
	Weaner 2	10	2	+	+	-	+	-	-	+	+	+	+	-	-	-	-	+	-	-
	Finisher	15	3	+	+	-	+	-	-	+	+	+	+	-	-	-	-	+	-	-
	Finisher	19	4	+	-	-	+	-	-	+	-	-	-	-	-	-	-	+	-	-
	Slaughter	24	5	+	ns	ns	+	ns	ns	+	-	ns	ns	-	-	ns	ns	+	ns	ns
D	Weaner 1	5	1	+	-	-	+	-	+	+	-	-	-	-	-	-	-	+	+	+
	Weaner 2	10	2	+	-	-	+	-	-	+	+	+	+	-	-	-	-	+	-	-
	Finisher	15	3	+	-	-	+	-	-	+	-	-	-	-	-	-	-	+	-	-
	Finisher	19	4	+	-	-	+	-	+	+	-	-	-	-	-	-	-	+	-	-
	Slaughter	24	5	+	ns	ns	+	ns	ns	+	-	ns	ns	-	-	ns	ns	+	ns	ns
E	Weaner 1	5	1	ns	ns	ns	ns	ns	ns	ns	ns	ns	ns	ns	ns	ns	ns	ns	ns	ns
	Weaner 2	10	2	+	-	-	+	-	-	-	-	-	-	-	-	-	-	+	+	-
	Finisher	15	3	+	-	-	+	-	+	-	-	-	-	-	-	-	-	+	-	-
	Finisher	19	4	+	+	+	+	-	+	-	-	-	-	-	-	-	-	+	-	-
	Slaughter	24	5	+	ns	ns	+	ns	ns	-	-	ns	ns	-	-	ns	ns	+	ns	ns
F	Weaner 1	5	1	+	+	-	+	-	-	-	-	-	-	-	-	-	-	+	-	-
	Weaner 2	10	2	+	-	-	+	-	-	-	-	-	-	-	-	-	-	+	-	-
	Finisher	15	3	+	-	-	+	-	+	-	-	-	-	-	-	-	-	+	-	-
	Finisher	19	4	+	+	-	+	-	+	-	-	-	-	-	-	-	-	+	-	-
	Slaughter	24	5	+	ns	ns	+	ns	ns	-	-	ns	ns	-	-	ns	ns	+	ns	ns
G	Weaner 1	5	1	+	ns	+	-	ns	-	-	-	ns	-	-	-	ns	ns	+	ns	-
	Weaner 2	10	2	+	+	+	-	-	-	-	-	-	-	-	-	-	-	-	-	-
	Finisher	15	3	+	-	-	-	-	-	-	-	-	-	-	-	-	-	-	-	-
	Finisher	19	4	+	-	-	-	-	-	-	-	-	-	-	-	-	-	-	-	-
	Slaughter	24	5	+	ns	ns	-	ns	ns	-	-	ns	ns	-	-	ns	ns	-	ns	ns

Herd pathogen status by herd, time-point (t/p) and sample matrix (serum, nasal swabs and oral fluids) throughout pig production (weaner and finishers) and at slaughter across 7 Irish farrow-to-finish herds using enzyme-linked immunosorbent assay (ELISA) and polymerase chain reaction (PCR) methodologies for four targeted respiratory pathogens [*Actinobacillus pleuropneumonia *(App), *Mycoplasma pleuropneumonia *(Mhyo), porcine reproductive and respiratory syndrome virus (PRRSV-1; European species and PRRSV-2; North American species) and influenza A virus (IAV). Positive (+) and negative (-) symbols indicate herd health status. Oral fluids and nasal swabs collected at t/p’s 1-4; serum at all t/p’s (1-5). No samples (ns) were collected/available from Herd E at t/p1 and nasal samples from Herd G at t/p 1

### Diagnostic evaluation

A comprehensive comparative analysis of diagnostic performance across different sample matrices and detection methods is presented in Table 6, including McNemar test results, Cohen’s kappa values and corresponding agreement interpretations for all four respiratory pathogens investigated. The diagnostic concordance between serological detection of pathogen-specific antibodies and PCR-based pathogen detection across all sample matrices demonstrated variable agreement levels, ranging from no agreement to fair agreement (κ = 0-0.307, McNemar *p* = < 0.001–0.003).

**Table 6:** Diagnostic performance comparison across different sample matrices and detection methods for four targeted respiratory pathogens

		Targeted Respiratory Pathogens								
		App		Mhyo		PRRSV-1			IAV	
Sample matrix / technique	Methods evaluation	Nasal (PCR)	Oral fluid (PCR)	Serum (ELISA)	Oral fluid (PCR)	Serum (ELISA)	Serum (PCR)	Oral fluid (PCR)	Serum (ELISA)	Oral fluid (PCR)
Nasal	*p-*value^*a*^	-	0.004	< 0.001	0.023	0.003	0.617	1	< 0.001	0.48
(PCR)	κ-value^*b*^	-	0.177	0.01	0.165	0.259	0.57	0.752	0.046	0.629
	agreement^c^	-	Slight	Slight	Slight	Fair	Moderate	Substantial	Slight	Substantial
Serum	*p-*value^*a*^	0.001	< 0.001	-	< 0.001	-	0.001	0.003	-	< 0.001
(ELISA)	κ-value^*b*^	0	0	-	0.136	-	0.307	0.27	-	0.02
	agreement^*c*^	None	None	-	Slight	-	Fair	Fair	-	Slight
Serum	*p-*value^*a*^	-	-	-	-	-	-	0.617	-	-
(PCR)	κ-value^*b*^	-	-	-	-	-	-	0.575	-	-
	agreement^*c*^	-	-	-	-	-	-	Moderate	-	-

McNemar^*a*^ (*p*-value = < 0.05) and Cohen Kappa^*b*^ (κ) test results comparing enzyme-linked immunosorbent assay (ELISA) and polymerase chain reaction (PCR) methodologies across different sample matrices (serum, nasal swabs and oral fluids) for four targeted respiratory pathogens [54]

Inter-matrix agreement analysis revealed pathogen-specific patterns when comparing nasal swabs and oral fluid PCR results. For the bacterial pathogens App and Mhyo, only slight agreement was observed between nasal and oral fluid samples (App: κ = 0.177, McNemar *p* = 0.004; Mhyo: κ = 0.165, McNemar *p* = 0.023). In contrast, viral pathogen detection demonstrated higher agreement between nasal and oral fluid samples, with PRRSV-1 detection showing substantial agreement between these matrices (κ = 0.752, McNemar *p* = 1.000), while IAV detection similarly demonstrated substantial agreement (κ = 0.629, McNemar *p* = 0.480). The evaluation of PRRSV-1 RNA detection across different sample matrices revealed moderate agreement between PCR-positive serum samples and both nasal swabs (κ = 0.570, McNemar *p* = 0.617) and oral fluid samples (κ = 0.575, McNemar *p* = 0.617). These comparable kappa values suggest that serum, nasal swabs and oral fluids demonstrate similar diagnostic utility for PRRSV-1 detection, with the non-significant McNemar test results indicating no preferential detection in any particular sample type.

## Discussion

Distinct serological patterns were observed for each respiratory pathogen across the production cycle. The high concurrent detection of antibodies from all four respiratory pathogens (27.24% at pig level and rising to 64.71% at herd level) demonstrates the complex pathogen landscape present in Irish farrow-to-finish operations. This finding aligns with previous European studies and highlights the endemic nature of these respiratory pathogens in commercial pig production [8, 20]. The substantial increase from pig-level to herd-level prevalence (67–100% at slaughter) reflects the aggregated nature of pathogen exposure across production cohorts or, more likely, the persistence of vaccination-derived antibodies combined with natural exposure to circulating field strains. It supports the focus of herd-level management strategies rather than individual animal interventions.

Polymerase Chain Reaction detection patterns varied considerably between pathogens and sample types. The documented co-circulation of pathogens at herd-level, with concurrent nucleic acid detection occurring in 11.1% of oral fluid and 15.4% of nasal swab samples, has significant clinical implications. The presence of multiple pathogens simultaneously may amplify disease severity and duration, necessitating comprehensive surveillance approaches that can detect multiple agents concurrently [3, 4, 55, 56]. This finding supports previous research by Pomorska-Mól et al. [55] that observed that in the presence of App, IAV-like lesions, viral replication and nasal IAV shedding increased. Thacker et al. [56] highlighted the influence of Mhyo infections on the intensity and prolongation of PRRSV-induced pneumonia, demonstrating synergistic interactions between respiratory pathogens.

The significant differences observed between ELISA and PCR detection methods across all sample matrices confirm that these approaches provide complementary rather than redundant information. Advanced PCR technology detects pathogen presence, while ELISA reveals host immune responses, offering different temporal windows of infection status. This finding emphasises the importance of incorporating both molecular and serological methods in comprehensive surveillance programs to capture the full spectrum of pathogen dynamics within herds.

The gradual seroconversion pattern observed across Mhyo-vaccinated herds, with incremental seropositivity increasing with age, was predicted [37, 49, 57]. The high seropositivity (> 82%) at finisher and slaughter stages cannot be attributed to early vaccination protocols, but suggests ongoing pathogen recirculation of Mhyo, resulting from an active infection, despite vaccination [20, 58]. Vaccination of pigs for Mhyo was common practice within this study. Similar vaccination practices have been reported in other European studies [11, 59].

The suboptimal performance of nasal swabs for Mhyo detection (0.3% prevalence) aligns with the pathogen’s colonisation preference for the lower respiratory tract, as documented in previous studies [28]. This finding reinforces the importance of understanding pathogen biology when selecting sampling strategies [25, 26]. Similar to other field studies of live pigs, nasal swabs were found to be the least sensitive sampling method for Mhyo detection. The predominant detection of Mhyo at the latter finisher stage (24 weeks of age) is reflective of the increased pen-prevalence observed by Hernandez-Garcia et al. [35], which also reported PCR-positive animals at later t/p’s correlates, to the presence of severe enzootic-like lesion scores at slaughter and an active presence of Mhyo. While Mhyo detection results with oral fluids are better than nasal swabs, and the use of tracheal sampling would provide superior sensitivity [27], this highlights the challenges in Mhyo surveillance and suggests that multiple sampling approaches may be necessary for comprehensive monitoring.

The remarkably high early seroconversion for App (97.6% at pig-level at 5 weeks of age) provides strong evidence for pathogen circulation within sow populations and early piglet exposure. The high seroconversion during t/p 1 (5 weeks of age) is mirrored in other studies [37]. This finding, validated through Apx IV toxin detection by ELISA, differentiates between infected and vaccinated animals [20, 37], confirms that App infection occurs early in the production cycle [60] and suggests inadequate lactogenic immunity transfer. The maintenance of high seropositivity (> 94%) through to slaughter across most herds (6 of the 7 herds) indicates persistent pathogen pressure throughout the production cycle. The temporal differences in App detection between sample matrices, with nasal swabs peaking at 10 weeks of age (t/p 2) and oral fluids at 5 weeks of age (t/p 1), may reflect different stages of infection and pathogen shedding patterns, potentially due to differences in bacterial colonisation sites or sampling methodology sensitivity. The elevated rates of App observed in the early production stages likely correspond to the decline of maternal antibodies, creating an epidemiological situation where pigs that acquired early infection during nursery reach peak infectivity when commingling with uninfected pen-mates at weaning [60]. The absence of vaccination for this pathogen in this study reflects a large Irish cross-sectional study, which reported that this is common practice on farrow-to-finish herds [11].

The high IAV antibody herd prevalence (≥ 86%) maintained throughout the study period, combined with concentrated viral detection during early production stages (5 weeks of age), suggests a pattern of early exposure followed by protective immunity development. These findings are consistent with high herd seropositivity (62%) documented at slaughter [37], and with Spanish studies reporting high seropositivity (≥ 90%) for multiple IAV lineages (H1N1, H3N2 and H1N2) within the same production batch within herds [20] and supports the hypothesis of endemic circulation within production systems.

The substantial agreement between oral fluid and nasal swab detection, combined with the absence of detection during finisher stages (19 and 24 weeks of age), indicates that IAV surveillance should focus on early production phases. Similar results correspond to a wean-to-finish study by Hernandez-Garcia et al. [35] in which IAV nucleic acid was only detected between 5 and 11 weeks of age. Additionally, Decorte et al. [31] reported that influenza RNA can be detected from nasal swabs and oral fluid from IAV experimentally infected pigs, with detection from oral fluid possible for a prolonged duration (~ 21 DPI to 4 weeks; nasal swabs are negative at 7 DPI). The recommendation for family oral fluid collection (pooled oral fluid sample from the sow and her piglets) from farrowing rooms emerges as a logical extension of these findings, potentially capturing the critical early exposure period more effectively.

The progression from high early seropositivity (50–79%) in vaccinated herds, probably due to passive immunity from the vaccinated dams to > 95% at slaughter (24 weeks of age) demonstrates the complex interplay between vaccination, passive immunity and active infection. This indicates that vaccination-derived antibodies made a primary contribution to the observed seroprevalence. This pattern suggests that while vaccination may provide some protection, it does not prevent pathogen circulation or infection. Similar to these results, an extensive Spanish study [20] reported 89% of herds seropositive for PRRSV, with the within-herd seroprevalence reported between 76 and 100% at slaughter. The superior detection rates from oral fluids (14–36%) compared to nasal swabs (6–11%) for three of the 4 PRRSV-1 seropositive herds supports previous experimental findings [35] and highlights the value of oral fluid sampling for this pathogen. These findings suggest that viral respiratory pathogens are more consistently detected across different sample types, possibly reflecting their broader tropism and more uniform shedding patterns compared to bacterial pathogens. The non-significant McNemar test results for both viral pathogens indicate no systematic bias toward detection in sample type (IAV; oral fluids and nasal swabs and PRRSV-1; oral fluids, nasal swabs and serum). This finding supports the use of multiple sample matrices for viral pathogen surveillance, as each may capture different phases of shedding or provide complementary diagnostic information depending on the stage of infection and individual animal factors.

The extended PRRSV-1 detection period from oral fluids (10–19 weeks of age) compared to other matrices (nasal swabs and serum samples; 5–15 weeks of age) aligns with experimental studies showing prolonged viral shedding [35, 61, 62]. The lack of detection of PRRSV-1 RNA from serum at the finisher stages, yet antibody positive, also corresponds to other PRDC studies [35, 61]. This finding has important implications for surveillance program design and suggests that oral fluid sampling may provide a broader window for pathogen detection.

The comparative performance data demonstrate that no single sample matrix is optimal for all pathogens; however, oral fluids consistently performed as well as or better than alternative matrices across the pathogen panel studied. This finding, combined with the practical advantages of pen-based collection (reduced animal stress, improved handler safety and increased throughput), supports the adoption of oral fluid sampling as a primary surveillance tool. The pathogen-specific temporal patterns observed emphasise the importance of strategic sampling timing. Early production stages (5–10 weeks of age) appear critical for detecting acute infections, while later stages may be more suitable for assessing chronic or persistent infections. These findings support the implementation of sequential testing protocols that can capture disease dynamics across the production cycle.

The absence of dam antibody and pathogen status data represents a significant limitation in interpreting early piglet results and passive immunity transfer. Future studies should incorporate sow population monitoring to better understand vertical transmission patterns and optimise early detection strategies. The mixing of study pigs with others from the same production batch, while reflecting commercial reality, may have influenced oral fluid results due to the pooled nature of sampling. This limitation highlights the need for careful study design considerations when evaluating pen-based sampling approaches.

The longitudinal nature of this study provides critical insights for tackling the multifactorial nature of PRDC in commercial pig production. The temporal pathogen detection patterns documented in this study enable the development of targeted intervention strategies at critical production stages. The early detection of App (97.6% seropositive at 5 weeks of age) and IAV (peak detection at 5 weeks of age) indicates that the weaner period represents a critical window for pathogen exposure and potential intervention. This finding supports the implementation of enhanced biosecurity measures during early production stages, including rigorous cleaning and disinfection protocols between batches, as well as optimised vaccination timing. The implementation of fixed spatial pen sampling would be well-founded due to the differences of shedding between individual pigs and period length of sample positivity to optimise pathogen surveillance. Pen-level oral fluid sampling allows producers to implement management interventions before clinical disease manifestation, reducing PRDC economic impact through early antimicrobial intervention when clinically justified, environmental adjustments and implementation of cohort-specific management strategies.

The vaccination efficacy data presented, particularly for Mhyo, where seroconversion exceeded vaccination expectations, provides evidence for ongoing pathogen circulation despite current vaccination protocols. The high seroprevalence of App at the first stage is suggestive that App infection originated from the sow herd and the inclusion of App vaccination protocols should be considered. This finding suggests that vaccination timing, antigen selection or delivery methods may require optimisation. The longitudinal approach enables producers and veterinarians to monitor vaccine performance in real-time and adjust protocols based on serological and molecular evidence of pathogen circulation.

The demonstration that multiple pathogens circulate simultaneously supports the adoption of multivalent vaccination strategies and highlights the importance of comprehensive immunisation programs that address the full spectrum of PRDC-associated pathogens rather than focusing on individual agents. The co-circulation of multiple pathogens (11.1–15.4% concurrent detection) enables targeted antimicrobial therapy rather than broad-spectrum empirical treatment. This precision approach supports antimicrobial stewardship principles by reducing unnecessary drug use while maintaining therapeutic efficacy. The longitudinal surveillance data enables monitoring of treatment outcomes and pathogen persistence, allowing for evidence-based decisions regarding treatment duration and follow-up interventions. This approach is particularly relevant given increasing regulatory pressure to reduce antimicrobial use in livestock production and the growing concern about antimicrobial resistance.

Pathogen-specific temporal patterns provide actionable intelligence for product flow optimisation. The concentration of IAV in early production stages versus increased Mhyo detection during finishing suggests phase-specific management strategies. Superior oral fluid performance for PRRSV-1 detection during later stages (10–19 weeks of age) enables chronic infection monitoring, informing decisions about population management strategies to minimise the impact of persistent infections.

Routine oral fluid surveillance provides cost-effective PRDC management through simultaneous multi-pathogen monitoring from pooled samples. Early detection capabilities enable proactive management decisions, preventing clinical disease outbreaks and associated losses. The approach provides a framework for industry-wide monitoring, supporting regional disease control and benchmarking of herd health performance.

## Conclusions

This longitudinal study validates oral fluid sampling as an effective, practical alternative for respiratory pathogen surveillance in commercial pig production, directly addressing the complex management challenges associated with PRDC. The documented pathogen co-circulation patterns emphasise the need for comprehensive monitoring approaches that can detect multiple agents simultaneously, supporting the multifactorial nature of PRDC management.

The pathogen-specific temporal patterns provide valuable guidance for optimising surveillance timings and methodology, enabling targeted interventions at critical production stages. These findings support the implementation of proactive surveillance programs using oral fluid sampling as a primary tool for data-driven herd health management decisions, ultimately contributing to improved PRDC control, reduced antimicrobial use and enhanced production efficiency in the pig industry.

## Data Availability

The datasets used for the results in this study are available from the corresponding author upon a reasonable request.

## Abbreviations

AIAO: all-in/all-out
App: *Actinobacillus pleuropneumonia*
CI: Confidence interval
Ct: Cycle threshold
DPI: Days post-infection
ELISA: Enzyme-linked immunosorbent assay
Κ: Cohen’s kappa
Mhyo: *Mycoplasma hyopneumoniae*
IAV: Influenza A virus
PCR: Polymerase chain reaction
PRDC: Porcine respiratory disease complex
PRRSV: Porcine reproductive and respiratory syndrome virus
PRRSV-1: Porcine reproductive and respiratory syndrome virus (European strain)
PRRSV-2: Porcine reproductive and respiratory syndrome virus (North American strain)
qRT-PCR: Real-time, quantitative reverse transcriptase polymerase chain reaction
RPM: Revolutions per minute
t/p: Time-point
UK: United Kingdom
